# The Associations between the Duration of Folic Acid Supplementation, Gestational Diabetes Mellitus, and Adverse Birth Outcomes based on a Birth Cohort

**DOI:** 10.3390/ijerph16224511

**Published:** 2019-11-15

**Authors:** Gang Cheng, Tingting Sha, Xiao Gao, Qiong He, Xialing Wu, Qianling Tian, Fan Yang, Cai Tang, Xihong Wu, Qunhui Xie, Yan Yan

**Affiliations:** Department of Epidemiology and Health Statistics, Xiangya School of Public Health, Central South University, 110 Xiangya Road, Kaifu District, Changsha 410078, Hunan, China; gangcheng@csu.edu.cn (G.C.); tingtingsha@csu.edu.cn (T.S.); 166901003@csu.edu.cn (X.G.); qqniuniu0525@163.com (Q.H.); wuxl9404@163.com (X.W.); 15274979382@163.com (Q.T.); 176912057@csu.edu.cn (F.Y.); 186911018@csu.edu.cn (C.T.); wuxihong@csu.edu.cn (X.W.); xiequnan@163.com (Q.X.)

**Keywords:** folic acid, gestational diabetes mellitus, cesarean delivery, macrosomia

## Abstract

This study aimed to examine the associations between the duration of folic acid (FA) supplementation, gestational diabetes mellitus (GDM), and adverse birth outcomes. A total of 950 mother-offspring pairs participated in the cohort study during 2015 in Changsha, China. The data were collected through home visits and perfected by maternal and child healthcare handbooks. Generalized linear models and stratified analyses were used for statistical analyses. The incidence of GDM in our cohort was 10.2%. FA supplementation for ≥3 months before pregnancy was associated with an increased risk of GDM (adjusted relative risk (aRR): 1.72; 95% CI: 1.17–2.53) and decreased risk of small-for-gestational-age (SGA) birth (aRR: 0.40; 95% CI: 0.18–0.88). In the group of FA supplementation for ≥3 months during pregnancy, GDM was associated with an increased risk of cesarean delivery (aRR: 1.36; 95% CI: 1.06–1.75) and macrosomia (aRR: 2.11; 95% CI: 1.06, 4.20), but the aRRs were lower than the RR_MH_ 1.53 (95% CI: 1.01–2.34) and 2.43 (95% CI: 1.27–4.66). Our study suggested that the longer duration of FA supplementation before pregnancy might increase the risk of GDM, but decrease the risk of SGA birth. Longer duration of FA supplementation during pregnancy had beneficial effects on birth outcomes in women with GDM. Further studies should consider a larger sample size to confirm these findings.

## 1. Introduction

Folic acid (FA) is a water-soluble B vitamin. During pregnancy, FA supplementation is required to increase the expansion of maternal blood volume and growth of the fetus [1]. In addition, FA supplementation before, and during, pregnancy can prevent the occurrence of neural tube defects (NTDs) [2]. From 2016, World Health Organization (WHO) recommends that pregnant women should take FA supplements at 400 µg/day, ideally before pregnancy, in order to prevent maternal anemia, puerperal sepsis, low birth weight (LBW), and pre-term birth [3]. On the basis of WHO guidelines, FA supplementation is included in standard antenatal care in many countries, and mandatory food FA fortification has been introduced in more than 80 countries [4]. So far, daily FA supplementation for pregnant women has become widespread around the world and FA supplementation rate is rising gradually.

However, recent evidence has revealed the associations between FA supplementation and gestational diabetes mellitus (GDM) among pregnant women. In a Chinese cohort study (*n* = 3474), daily FA supplementation, during early pregnancy, increased the risk of GDM [5]. Moreover, a prospective study in China (*n* = 326) showed that women who did not take FA and took FA for > 90 days had a higher incidence of GDM compared with those who took FA for ≤60 days [6]. Another Chinese cohort study (*n* = 4353) showed that FA supplement use ≥800 µg/day from pre-conceptional ≥4 weeks until ≥16 weeks during pregnancy was associated with higher GDM risk compared with non-users [7]. Due to the limited number of studies, the association between daily FA supplementation and GDM has been controversial at present.

According to the latest data from the United Nations International Children’s Emergency Fund, one of the leading causes of death among children under age 5 in 2016 was pre-term birth complications, which accounted for about 18% of all causes of death [8]. Pre-term birth and other adverse birth outcomes need to be paid global attention. At present, lots of researches have indicated that GDM is associated with increased risks of adverse birth outcomes, including cesarean delivery, pre-term birth, macrosomia, and large-for-gestational-age (LGA) birth [9,10]. In addition, the literature has provided evidence of the protective effect of FA supplementation on adverse birth outcomes, including pre-term birth, small-for-gestational-age (SGA) birth, and LBW [11,12,13]. It was contradictory that FA supplementation increased the risk of GDM, and GDM increased the risks of adverse birth outcomes, then FA supplementation decreased the risks of adverse birth outcomes. A cohort study showed that continued FA supplementation with 400 µg/day after the first trimesters of pregnancy significantly increased the risk of LGA birth [14]. The above causal relationship is logical if the longer duration of FA supplementation leads to an increased risk of GDM. But it is uncertain whether the longer duration of FA supplementation increases the adverse effect of GDM on adverse birth outcomes. 

Considering that the neural tube closes by day 28 of pregnancy, and the critical periods for anencephaly, and spina bifida, respectively occur between days 35 to 40, and days 37 to 42, FA supplementation should start before the first month of pregnancy [15]. Kinetic studies suggest that it takes at least 12 weeks with 400 μg/day FA supplementation to achieve adequate plasma FA concentrations, which are associated with reduced risk of NTD [16]. At present, the WHO and most countries recommend a FA supplement of 400 µg/day from pre-conceptional 4 to 12 weeks until 8 to 12 weeks during pregnancy [17]. Therefore, this article focused on the associations between FA supplementation for ≥3 months before, and during, pregnancy, and GDM and adverse birth outcomes, compared with FA supplementation for <3 months.

There is currently no conclusive evidence regarding the associations between the duration of FA supplementation, GDM, and adverse birth outcomes. This study aimed to examine the effects of duration of FA supplementation before, and during, pregnancy on GDM and adverse birth outcomes. Furthermore, this study aimed to estimate the risks of GDM stratified by duration of FA supplementation on adverse birth outcomes. This study was intended to explore the potentially deleterious effects of long-term duration of FA supplementation on GDM and adverse birth outcomes, in order to provide favorable evidence for antenatal nutritional interventions.

## 2. Materials and Methods 

### 2.1. Study Design and Sample

This study was based on a prospective birth cohort study that began in 2015 and was conducted in three communities in the Kaifu District of Changsha City, Hunan Province, China. The three communities were randomly selected as our study sites. From January 2015 to December 2015, 1,286 pregnant women had live births at our study sites, and registered their birth information in the Community Health Management Information System (CHMIS). These mother-offspring pairs were recruited for the cohort. The cohort study was approved by the Independent Ethics Committee of Clinical Pharmacology Institute, Central South University, Changsha, China (Project number: CTXY-130041-3-2). Additional details of the cohort study were published previously [18]. The inclusion criteria for our study included: (1) Infants had health care records in the CHMIS; (2) mothers and infants’ guardians agreed to participate and signed the written informed consents; and (3) mothers took FA supplements at a dose of 400 µg/day before, and during, pregnancy. The exclusion criteria included: (1) Mothers had a history of mental illness or brain disease; (2) mothers had diabetes before pregnancy; and (3) infants were multiple births or had any congenital diseases.

### 2.2. Data Collection and Procedure

Trained investigators used a self-designed questionnaire with 60-items to interview mothers by means of home visits. Information about sociodemographic, maternal nutritional, lifestyle, medical factors, and neonatal anthropometric measures were collected through home visits at 1 month postpartum. The records from CHMIS, and maternal and child healthcare handbooks, which recorded the results of the antenatal examination and neonatal birth information, were used for checking and supplementing. The calculation of the sample size was based on the requirements for the cohort studies of disease [19]. According to the previous study [5], the estimated incidence of GDM among the FA supplementation group, and non-FA supplementation group, respectively were 3.96% and 8.86%. Assuming that we considered a two-sided test with α = 0.05 and β = 0.15, the total sample size required for the study was 888.

### 2.3. Measurement of Duration of FA Supplementation

Information on the use of FA supplements was provided by questioning during the interview, including the initiation and ending time of FA supplementation (e.g., Have you ever taken FA supplements or FA-containing multivitamins before, and during, pregnancy? When did you start to take FA supplements or FA-containing multivitamins? When did you stop to take FA supplements or FA-containing multivitamins?), dose, frequency of use, and supplements brand name. Here FA supplementation included taking exclusive FA supplements and FA-containing multivitamins. The duration of FA supplementation was classified into two groups: <3 months and ≥3 months [16,17]. Not taking FA supplements was included in the group of FA supplementation for <3 months. 

### 2.4. Measurement of GDM

Pregnant women underwent a fasting blood glucose test or 75 g oral glucose tolerance test (OGTT) between 24 and 28 weeks of gestation. GDM was diagnosed according to the International Association of Diabetes and Pregnancy Study Groups criteria [20], if pregnant women met one of the followings: (1) ≥5.1 mmol/L of fasting plasma glucose concentrations; (2) ≥10.0 mmol/L of plasma glucose concentrations 1-h post-OGTT; (3) ≥8.5 mmol/L of plasma glucose concentrations 2-h post-OGTT. The investigators identified whether pregnant women were diagnosed as GDM by obtaining their plasma glucose concentrations from CHMIS and maternal healthcare handbooks.

### 2.5. Measurement of Infant Birth Outcomes

The adverse birth outcomes included cesarean delivery, pre-term birth, early term birth, macrosomia, LBW birth, LGA birth, and SGA birth. Pre-term birth was defined as delivery before 37 gestational weeks, and early term birth as delivery before 39 gestational weeks. The gestational week was calculated from the first day of the last normal menstrual period. Macrosomia was defined as birth weight ≥4000 g and LBW birth as <2500 g. LGA birth was determined as birth weight above the 90th percentile for a specific gestational age, and SGA birth as below the 10th percentile, based on a global reference for fetal-weight and birthweight percentiles, published in 2011 [21]. Birth weight was tested twice by trained nurses on an electronic scale and read to the nearest 0.1 kg, excluding the weight of clothes, shoes, and caps.

### 2.6. Measurement of Covariates

The potential confounding variables were identified from previous studies [5,6,7,12]. Sociodemographic factors included maternal age at pregnancy (<25, 25–29, 30–34, ≥35 years old) [14], maternal education (≤junior high school, senior high school, ≥college degree) [6,12], and monthly household income (≤2000, 2001–5000, > 5000 CNY) [18]. Gestational characteristics included postpartum body mass index (BMI) (<18.5, 18.5–23.9, 24–27.9, ≥28 kg/m^2^), parity (primiparous or multiparous), iron supplementation (yes or no), multivitamin supplementation (yes or no), gestational dietary intake (≥3 times a week or <3 times a week), passive smoking (yes or no), alcohol use (yes or no), gestational hypertension (yes or no), and gestational anemia (yes or no). BMI was categorized into four levels: <18.5 kg/m^2^ (underweight), 18.5–23.9 kg/m^2^ (normal weight), 24–27.9 kg/m^2^ (overweight), and 28 kg/m^2^ (obese), which was based on the recommendations from the Working Group on Obesity in China [22]. BMI was calculated as weight (kg) divided by height (m) squared. Maternal height and postpartum weight were obtained from the records in CHMIS and maternal healthcare handbooks. Gestational dietary intake was measured by the frequency of eating meat or liver, fish or seafood, egg, milk products, and fruits or vegetables [18]. Passive smoking was measured by asking mothers “Were you exposed to second-hand smoke for over 15 min a day?’’ [18]. Alcohol use was measured by asking mothers “Did you drink any alcohol during your last pregnancy?” [18]. Gestational hypertension was diagnosed as systolic blood pressure (BP) ≥140 mm Hg or diastolic BP ≥90 mm Hg after the 20th gestational week, regardless of proteinuria [23]. Gestational anemia was defined as hemoglobin less than 110 g/L during pregnancy according to the WHO criteria [24]. The family history of diabetes (yes or no), defined as that one of the grandparents had been diagnosed with diabetes, and infant gender (male or female) were also included.

### 2.7. Statistical Analysis

The data were presented as frequency and percentage, since all variables and outcomes were categorical variables. The differences in maternal characteristics between two groups of the duration of FA supplementation (<3 months and ≥3 months) were compared using the chi-square test. The differences in infant characteristics between GDM and non-GDM group were also compared using the chi-square test.

Generalized linear models were used to assess the risk of FA supplementation for ≥3 months on GDM, compared with FA supplementation for <3 months. The distribution was specified as “binomial” and the link function as “log”. Relative risk (RR) and corresponding 95% confidence intervals (CIs) quantified the extent of risks. The potential confounders included maternal age, education, average monthly household income, postpartum BMI, parity, passive smoking, family history of diabetes, iron supplementation, multivitamin supplementation, gestational dietary intake, and alcohol use. We assessed the interaction between the duration of FA supplementation and multivitamin supplementation on GDM [25]. Generalized linear models were also used to assess the effects of GDM on adverse birth outcomes compared with non-GDM, as well as the effects of FA supplementation for <3 months on adverse birth outcomes. Here, the potential confounders in the adjusted analyses included maternal age, education, average monthly household income, postpartum BMI, parity, passive smoking, family history of diabetes, iron supplementation, multivitamin supplementation, gestational dietary intake, alcohol use, gestational hypertension, gestational anemia, and infant gender. We assessed the interactions between GDM and the duration of FA supplementation on adverse birth outcomes.

The stratified analyses were conducted to identify whether the duration of FA supplementation was a confounder, or an effect modifier in the associations between GDM and adverse birth outcomes. The Breslow-Day test was used to assess the homogeneity of the RRs in the two duration groups of FA supplementation. The Mantel-Haenszel chi-square test was used to estimate the associations between GDM and adverse birth outcomes, after adjusting for the duration of FA supplementation. It can compute a weighted average of the RRs across the two duration groups of FA supplementation.

The missing values were estimated by the multiple imputation methods, which can reduce bias and increase precision and is recommended as a routine practice [26]. A two-tailed *p*-value < 0.05 was regarded as statistically significant. All statistical analyses were performed using IBM SPSS Statistics, version 22.0 (IBM, New York, NY, USA).

## 3. Results

### 3.1. Participant Characteristics

Our study included 950 mother-offspring pairs, after excluding 265 infants who did not have health care records in the CHMIS, 45 mothers who refused to participate, 25 multiple births, and 1 mother who had diabetes before pregnancy.

Table 1 shows maternal characteristics according to the duration of FA supplementation. In our birth cohort, the incidence of FA supplementation was 98.6%. The rate of FA supplementation during pregnancy was higher (97.9%) than that before pregnancy (65.3%). The rate of FA supplementation for ≥3 months before pregnancy was 30.8%, and that during pregnancy was 73.9%. Women who were more highly educated (*p* = 0.008) and took iron supplements (*p* = 0.008 and *p* = 0.003) were more likely to take FA supplements for ≥3 months both before, and during, pregnancy. In addition, women who had a higher monthly income (*p* = 0.005) were more likely to take FA supplements for ≥3 months during pregnancy. Women who used multivitamin supplements (*p* = 0.004) were more likely to take FA supplements for ≥3 months before pregnancy. The sample with missing data and those estimated by multiple imputations had similar maternal characteristics according to the duration of FA supplementation (see Supplemental Table S1).

Table 2 shows the infant characteristics between groups of GDM and non-GDM. The incidence of GDM was 10.2%. The rates of cesarean delivery, pre-term birth, early term birth, macrosomia, LBW birth, LGA birth, and SGA birth in our cohort were 39.1%, 3.8%, 29.1%, 6.7%, 2.8%, 21.2%, and 5.1%, respectively. Compared with non-GDM, pregnant women with GDM were more likely to have a cesarean delivery (*p* = 0.045) and macrosomia (*p* = 0.006). The rates of infant gender, pre-term birth, early term birth, LBW birth, LGA birth, and SGA birth, were similar between GDM and non-GDM group.

### 3.2. Associations between Duration of FA Supplementation and GDM

Table 3 shows the associations of the duration of FA supplementation and GDM. Compared with FA supplementation for <3 months, FA supplementation for ≥3 months before pregnancy was associated with an increased risk of GDM with crude RR (cRR) 1.71 (95% CI: 1.17–2.50) and adjusted RR (aRR) 1.72 (95% CI: 1.17–2.53). However, FA supplementation for ≥3 months during pregnancy was not significantly associated with GDM.

### 3.3. Associations of GDM and Duration of FA Supplementation with Adverse Birth Outcomes

Table 4 shows the effects of GDM and the duration of FA supplementation on adverse birth outcomes. In the multivariate analyses not adjusting for the duration of FA supplementation, GDM was significantly associated with an increased risk of cesarean delivery (aRR: 1.30; 95% CI: 1.03–1.63) and macrosomia (aRR: 2.11; 95% CI: 1.16–3. 83). The associations remained significant after adjusting for the duration of FA supplementation before or during pregnancy, and the aRRs were similar to cRR. We did not observe that GDM was significantly associated with pre-term birth, early term birth, LBW birth, LGA birth, and SGA birth. Only the association between duration of FA supplementation before pregnancy and SGA birth was observed (aRR: 0.40; 95% CI: 0.18–0.88). We observed the significant interactions between GDM and duration of FA supplementation before pregnancy on cesarean delivery (aRR: 1.38; 95% CI: 1.01–1.89) and macrosomia (aRR: 2.45; 95% CI: 1.08–5.57). In addition, we observed the significant interactions between GDM and duration of FA supplementation during pregnancy on macrosomia (aRR: 2.50; 95%CI: 1.10, 5.68).

Table 5 shows the associations between GDM and adverse birth outcomes stratified by duration of FA supplementation. In the analyses stratified by duration of FA supplementation during pregnancy, GDM was associated with an increased risk of cesarean delivery with RR_MH_ 1.53 (95% CI: 1.01–2.34), which was higher than the cRR (1.28; 95% CI: 1.02–1.59). In the group of FA supplementation for ≥3 months during pregnancy, GDM was associated with an increased risk of cesarean delivery with aRR 1.36 (95% CI: 1.06–1.75), which was lower than RR_MH_.

In the analyses stratified by duration of FA supplementation before pregnancy and during pregnancy, GDM was associated with an increased risk of macrosomia with RR_MH_ 2.46 (95% CI: 1.28–4.73) and 2.43 (95% CI: 1.27–4.66), which were both higher than the cRR 2.24 (95% CI: 1.27–3.97). In the group of FA supplementation for ≥3 months before pregnancy, GDM was associated with an increased risk of macrosomia with cRR 2.56 (95% CI: 1.04–6.29). But the association was not statistically significant in the adjusted analysis. Likewise, in the group of FA supplementation for ≥3 months during pregnancy, GDM was associated with an increased risk of macrosomia with aRR 2.11 (95% CI: 1.06, 4.20), which was lower than RR_MH_. In the analyses stratified by duration of FA supplementation, we did not observe that GDM was significantly associated with pre-term birth, early term birth, LBW birth, LGA birth, or SGA birth.

## 4. Discussion

Our study suggested that FA supplementation for ≥3 months before pregnancy was associated with a higher risk of GDM and lower risk of SGA birth, but during pregnancy was not. FA supplementation for ≥3 months, during pregnancy, might decrease the effect of GDM on cesarean delivery and macrosomia. These associations remained statistically significant after adjusting for potential confounders.

In our cohort, the FA supplementation rate was 98.6%. A high FA supplementation rate among Chinese women has previously been reported. In 2015, approximately 93.1% of 1,921 pregnancy women used FA supplementation in Tianjin, China [27]. Our study found that the FA supplementation rate, in the first trimester of pregnancy, was 73.9%. In the previous study, 75.6 % of rural women from 220 counties in China reported taking FA supplements in the first trimester [28], which was similar to our finding. In our study, 30.8% of participants took FA supplementation for ≥3 months before pregnancy, which was more than that (22.4%) in a cross-sectional study from 2015 to 2016 [27]. The rate in our study is higher possibly because all of our participants come from the city. The incidence of GDM in our cohort was 10.2%, which approached the total rate in mainland China (14.8%) [29]. Compared with 6.8% in 2008 [30], the rate of GDM among Chinese women was on the rise. The GDM rate in China was close to the overall rate (11.1%) in developed countries of Europe during 2010-2016 [31]. Similar to the record of China from the WHO (34.9%) [32], the rate of cesarean delivery in our cohort was 39.1%. It approached the rates in some developed countries, such as America (32.0%), Australia (33.3%), and Italy (35.5%) [33]. The incidence of macrosomia in our cohort was 6.7%, which is similar to the result of a multicenter survey in China (7.3%) [34]. The macrosomia rate in China remains higher than in most developing countries [35].

Our results showed that FA supplementation for ≥3 months before pregnancy increased the risk of GDM, compared with FA supplementation for <3 months. In another Chinese study [6], women who took FA for 3 months had a higher incidence of GDM compared with those who took FA for ≤2months. Our study discussed, respectively, the effects of the duration of FA supplementation before, and during, pregnancy on GDM. However, this study focused on the total duration from pre-pregnancy to early pregnancy. Similarly, both this and our study showed the tendency that, a longer duration of FA supplementation was significantly associated with an increased risk of GDM. Several studies have reported the association between the use of FA supplements and GDM. A Danish cross-sectional study showed no clear association between pre-conceptional FA use and the diagnosis of diabetes [36]. On the contrary, a Chinese cohort study found that daily FA use in the first trimester increased the risk of GDM [5]. In the Danish study, 62.1% of pregnancy planners used FA, including FA exclusive supplements, multivitamins exclusive supplements, and FA and multivitamins supplements. However, the Chinese study excluded the multivitamins user. Thus, the Danish study examined the effect of pre-conceptional FA and multivitamins use on GDM, not only the FA supplementation. It was likely that the overestimation of FA meaningfully distorts the association between FA supplementation and GDM. Furthermore, several studies have reported the association between the dosage of FA supplements and GDM. Another Chinese cohort reported that FA supplement use ≥800 µg/day from ≥4 weeks before pregnancy to ≥16 weeks during pregnancy was related to elevated GDM risk, compared with never using any FA supplement or using FA with a daily dose <400 µg or for <4 weeks [7]. The mechanism underlying the adverse effect of longer duration of FA supplementation on GDM remains unclear. One possible explanation may be related to the accumulated concentration of plasma FA. Recent studies revealed that the high levels of unmetabolized FA could adversely modulate natural killer cells. Then, dampened natural killer responses to infections would result in the infiltration of beta-cell in type 1 diabetics [37].

In our study, we found that FA supplementation for ≥3 months before pregnancy decreased the risk of SGA birth. Two cohort studies and one literature review both showed that pre-conceptional FA supplementation was significantly associated with a reduced risk of SGA birth, compared with no FA supplementation [11,12,38]. A cohort study showed that continued FA supplementation with 400 µg/day after the first trimesters of pregnancy significantly increased the risk of LGA birth [14]. At present, there is no direct evidence supporting the association between the duration of FA supplementation before pregnancy and SGA. The longer duration of FA supplementation before pregnancy may contribute to a higher birth weight. The exact mechanisms remain poorly understood. One explanation is that FA is an important methyl-group vitamin, and maternal plasma FA levels impact offspring DNA methylation [39], which relates to fetal weight [40]. Another explanation is that FA as a methylating micronutrient increases fetal weight by influencing hepatic gene expression and IGF signaling [14].

Our results showed that FA supplementation for ≥3 months during pregnancy might decrease the effect of GDM on cesarean delivery. The reviewed literature suggested that multiple-micronutrient supplementation, with iron and FA, was not associated with cesarean delivery [41]. So far, there is not enough evidence to support that FA supplementation is associated with cesarean delivery. Moreover, based on the multivariate analysis in our study (see Table 4), FA supplementation for ≥3 months before and during pregnancy did not increase the risk of cesarean delivery. A larger-sample cohort observed that the risk of cesarean delivery was higher in women with GDM than those without diabetes [9]. We assumed that FA supplementation may cause indirect effects on cesarean delivery by the complicated mechanism. There was mounting evidence that inadequate maternal FA status during pregnancy may lead to low infant birth weight [13]. Compared with normal birth weights, mothers of neonates with extremes of birth weights (<2500 g or >4000 g) had increased risks of having a cesarean delivery [42]. Adequate FA supplementation can lead to a more normal birthweight, thus, indirectly reducing the rate of cesarean delivery.

In our study, we found that FA supplementation for ≥3 months during pregnancy might decrease the effect of GDM on macrosomia. In a birth cohort study, the continued FA supplementation with 400 µg/day, in the second and third trimesters of pregnancy, significantly increased the risk of LGA birth, compared with FA supplementation only in the first trimester [14]. But it did not significantly increase the risk of LBW and macrosomia [14]. At present, there is no direct evidence to support the fact that FA supplementation, for ≥3 months during pregnancy, increases the risk of macrosomia. A cohort study showed that a high dosage of FA (≥1000 µg/day) may be associated with an increased risk of SGA at birth, compared with not taking FA [43]. In general, infants with SGA weigh below the tenth percentile for the gestational age at birth. Therefore, infants weighed less at birth when their mothers took a high dosage of FA. We presumed that continuing FA supplementation after the first trimester of pregnancy increased cumulative FA intake [14], which was probably related to lower birth weight and the decreased risk of macrosomia. GDM is a known independent risk factor for fetal macrosomia [9]. In our study, we observed the significant interactions between GDM and the duration of FA supplementation during pregnancy on macrosomia. One assumption is that the negative effect of FA supplementation, for ≥3 months during pregnancy on macrosomia, may offset a part of the positive effect of GDM on macrosomia.

The biological mechanism by which FA supplementation for ≥3 months during pregnancy may decrease the effect of GDM on cesarean delivery and macrosomia is unclear. The main explanation focuses on the relationship of FA supplementation and GDM on birth weight. We make the following two hypotheses. First, continuing FA after the first trimester of pregnancy can increase maternal and cord blood folate status and lead to a lower homocysteine concentration [44], which is associated with decreased birth weight [45]. Homocysteine levels are significantly elevated among women with GDM, compared with those without GDM [46]. Acute homocysteine exposure shows detrimental effects on pancreatic β-cell metabolism and insulin secretion [47]. Therefore, continuous FA supplementation is not conducive to the occurrence of GDM and is associated with lower birth weight. Secondly, FA provides methyl groups for biochemical reactions in the one-carbon metabolism pathway [48], and maternal plasma FA levels impact DNA methylation in newborns [39]. Therefore, neonatal DNA methylation is related to birth weight [40]. The candidate genes for fetal programming displays significant methylation differences between GDM and cesarean-GDM groups [49]. Intrauterine exposure to GDM has long-lasting effects on offspring, possibly mediated by DNA methylation [50]. Therefore, both FA and GDM may affect the growth of offspring through DNA methylation.

In our study, we did not observe that FA supplementation for ≥3 months before pregnancy significantly decreased the effects of GDM on any adverse birth outcomes after adjusting for potential confounders. There are two possible explanations in this case. Firstly, the sample size and the number of positive results in our study is not large enough to reveal the true effects of GDM on adverse birth outcomes, stratified by the duration of FA supplementation. Secondly, the duration of FA supplementation before pregnancy did not actually affect the relationships between GDM and adverse birth outcomes. At present, little is known about the effects of duration of FA supplementation before pregnancy on GDM and infant birth outcomes. In a prospective cohort study, pre-conceptional FA supplementation was inversely associated with the risk of pre-term birth and SGA birth [12]. A cohort study showed that pre-conceptional FA supplementation was associated with higher birth weight and a reduced risk of LBW and SGA birth, compared with no FA supplementation [38]. A literature review showed that FA supplementation before pregnancy was significantly associated with a reduced risk of SGA birth, but not associated with pre-term delivery [11]. A cohort study showed that pre-conceptional FA supplementation for ≥1 year was associated with a decreased risk of spontaneous pre-term delivery, compared with no supplementation [51]. More research is required to clarify the associations between the duration of FA supplementation before pregnancy and maternal gestational diseases and fetal growth.

Our study has several strengths. First, the study was a prospective birth cohort, which decreased the influence of selection or recall bias. We excluded mothers with mental illnesses or brain diseases and infants with multiple births, avoiding information bias. We used maternal and child healthcare handbooks to check and supplement the collected data, minimizing the risks of recall bias. Second, we collected enough data, including all kinds of confounding factors in the adjusted analyses, obtaining the real effect of duration of FA supplementation on GDM and adverse birth outcomes. Third, the incidences of FA supplementation, GDM, cesarean delivery, and macrosomia in our study were almost consistent with other regions in China [27,28,29,32,33,34]. It meant that our result could apply to populations from other regions in China.

Several limitations are present in this study. First, the known associations between GDM and other adverse birth outcomes, such as pre-term birth and LGA birth, cannot be assessed possibly due to smaller sample size and insufficient power in our study. Second, we did not measure the serum concentration of FA before pregnancy or early in pregnancy. Higher FA level in serum was associated with a higher risk of GDM and lower risks of adverse birth outcomes [25,52]. Third, the total FA intake is unknown because we did not consider the FA intake from diet. Fourth, recall bias and response bias might exist because we collected information about maternal FA supplementation at 1 month after giving birth. Further studies should consider a larger sample size, the total duration from pre-conception to post-conception, and complicated statistical methods, such as path model analysis and the decision tree model to examine the associations between FA supplementation, GDM, and adverse birth outcomes.

## 5. Conclusions

The study clarified the potential risk of longer duration of FA supplementation, which is significance for pregnant women and fetuses. The longer duration of FA supplementation before pregnancy might increase the risk of GDM, but decrease the risk of SGA birth. Longer duration of FA supplementation during pregnancy had beneficial effects on birth outcomes in women with GDM. Further studies should consider a larger sample size to confirm these findings.

## Figures and Tables

**Table 1:** Maternal characteristics according to the duration of folic acid supplementation in the cohort (*n* = 950).

Characteristics	Before Pregnancy		*p*-Value	During Pregnancy		*p*-Value
	<3 Months (*n* = 657)	≥3 Months (*n* = 293)		<3 Months (*n* = 248)	≥3Months (*n* = 702)	
Age at pregnancy (years)			0.059			0.369
<25	35 (5.3)	11 (3.8)		13 (5.2)	33 (4.7)	
25–29	325 (49.5)	124 (42.3)		125 (50.4)	324 (46.2)	
30–34	223 (33.9)	112 (38.2)		76 (30.6)	259 (36.9)	
≥35	74 (11.3)	46 (15.7)		34 (13.7)	86 (12.3)	
Education			0.008 *			0.008 *
≤Junior high	29 (4.4)	4 (1.4)		15 (6.0)	18 (2.6)	
Senior high	91 (13.9)	28 (9.6)		38 (15.3)	81 (11.5)	
≥College	537 (81.7)	261 (89.1)		195 (78.6)	603 (85.9)	
Average monthly household income (CNY)			0.051			0.005 *
≤2000	22 (3.3)	14 (4.8)		12 (4.8)	24 (3.4)	
2001–5000	364 (55.4)	138 (47.1)		150 (60.5)	352 (50.1)	
>5000	271 (41.2)	141 (48.1)		86 (34.7)	326 (46.4)	
Postpartum BMI (kg/m^2^)			0.673			0.468
<18.5	13 (2.0)	5 (1.7)		7 (2.8)	11 (1.6)	
18.5–23.9	383 (58.3)	160 (54.6)		145 (58.5)	398 (56.7)	
24–27.9	207 (31.5)	99 (33.8)		73 (29.4)	233 (33.2)	
≥28	54 (8.2)	29 (9.9)		23 (9.3)	60 (8.5)	
Parity			0.326			0.396
Primiparous	450 (68.5)	210 (71.7)		167 (67.3)	493 (70.2)	
Multiparous	207 (31.5)	83 (28.3)		81 (32.7)	209 (29.8)	
Iron supplementation			0.008 *			0.003 *
Yes	394 (60.0)	202 (68.9)		136 (54.8)	460 (65.5)	
No	263 (40.0)	91 (31.1)		112 (45.2)	242 (34.5)	
Multivitamin supplementation			0.004 *			0.093
Yes	367 (55.9)	193 (65.9)		135 (54.4)	425 (60.5)	
No	290 (44.1)	100 (34.1)		113 (45.6)	277 (39.5)	
Family history of diabetes			0.473			0.155
Yes	29 (4.4)	10 (3.4)		14 (5.6)	25 (3.6)	
No	628 (95.6)	283 (96.6)		234 (94.4)	677 (96.4)	
Gestational hypertension			0.368			0.132
Yes	36 (5.5)	12 (4.1)		17 (6.9)	31 (4.4)	
No	621 (94.5)	281 (95.9)		231 (93.1)	671 (95.6)	
Gestational anemia			0.155			0.122
Yes	89 (13.5)	30 (10.2)		38 (15.3)	81 (11.5)	
No	568 (86.5)	263 (89.8)		210 (84.7)	621 (88.5)	

Data are presented as frequency (%). The chi-square test was used to compare proportions. Level of significance: * *p* < 0.01.

**Table 2:** Comparisons of infant characteristics between groups of gestational diabetes mellitus (GDM) and non-GDM in the cohort (*n* = 950).

Infant characteristics	Total	GDM (*n* = 97)	Non-GDM (*n* = 853)	*p*-Value
Infant gender				0.100
Male	493 (51.9)	58 (59.8)	435 (51.0)	
Female	457 (48.1)	39 (40.2)	418 (49.0)	
Cesarean delivery				0.045 *
Yes	371 (39.1)	47 (48.5)	324 (38.0)	
No	579 (60.9)	50 (51.5)	529 (62.0)	
Pre-term birth				0.705
Yes	36 (3.8)	3 (3.1)	33 (3.9)	
No	914 (96.2)	94 (96.9)	820 (96.1)	
Early term birth				0.966
Yes	276 (29.1)	28 (28.9)	248 (29.1)	
No	674 (70.9)	69 (71.1)	605 (70.9)	
Macrosomia				0.006 **
Yes	64 (6.7)	13 (13.4)	51 (6.0)	
No	886 (93.3)	84 (86.6)	802 (94.0)	
LBW birth				0.875
Yes	27 (2.8)	3 (3.1)	24 (2.8)	
No	923 (97.2)	94 (96.9)	829 (97.2)	
LGA birth				0.240
Yes	201 (21.2)	25 (25.8)	176 (20.6)	
No	749 (78.8)	72 (74.2)	677 (79.4)	
SGA birth				0.659
Yes	48 (5.1)	4 (4.1)	44 (5.2)	
No	902 (94.9)	93 (95.9)	809 (94.8)	

Abbreviations: GDM, gestational diabetes mellitus; LBW, low birth weight; LGA, large-for-gestational-age; SGA, small-for-gestational-age. Data are presented as frequency (%). The chi-square test was used to compare proportions. Level of significance: * *p* < 0.05, ** *p* < 0.01.

**Table 3:** Associations between duration of folic acid supplementation and gestational diabetes mellitus.

Variables	Model 1 ^a^	Model 2 ^b^	Model 3 ^c^	Model 4 ^d^	Model 5 ^e^
Before pregnancy					
<3 months	1.00	1.00	1.00	1.00	1.00
≥3 months	1.71 (1.17, 2.50) *	1.68 (1.14, 2.46) *	1.70 (1.16, 2.50) *	1.72 (1.17, 2.53) *	1.68 (0.90, 3.15)
During pregnancy					
<3 months	1.00	1.00	1.00	1.00	1.00
≥3 months	1.02 (0.66, 1.57)	1.04 (0.67, 1.60)	1.14 (0.73, 1.78)	1.16 (0.75, 1.81)	0.92 (0.52, 1.65)

Data are presented as relative risk and 95% confidence interval. Generalized linear models were used to assess the effects. Level of significance: * *p* < 0.01. ^a^ Model 1 did not adjust for anything. ^b^ Model 2 adjusted for maternal age, education, and average monthly household income. ^c^ Model 3 adjusted for Model 2 plus postpartum BMI, parity, passive smoking, and family history of diabetes. ^d^ Model 4 adjusted for Model 3 plus iron supplementation, multivitamin supplementation, gestational dietary intake, and alcohol use. ^e^ Model 5 assessed the interaction between the duration of folic acid supplementation and multivitamin supplementation on gestational diabetes mellitus.

**Table 4:** Effects of GDM and duration of folic acid (FA) supplementation on adverse birth outcomes.

Outcomes	Model 1 ^c^	Model 2 ^d^	Model 3 ^d^	Model 4 ^e^	Model 5 ^e^	Model 6 ^f^	Model 7 ^f^
Cesarean delivery							
cRR	1.28 (1.02, 1.59) *	1.27 (1.02, 1.59) *	1.28 (1.02, 1.59) *	1.03 (0.87, 1.22)	0.98 (0.82, 1.18)	1.38 (1.02, 1.88) *	1.26 (0.94, 1.68)
aRR ^a^	1.30 (1.03, 1.63) *	1.25 (1.01, 1.57) *	1.26 (1.01, 1.58) *	1.05 (0.89, 1.25)	1.02 (0.85, 1.21)	1.38 (1.01, 1.89) *	1.29 (0.97, 1.72)
Pre-term birth							
cRR	0.80 (0.25, 2.56)	0.82 (0.25, 2.62)	0.80 (0.25, 2.56)	0.87 (0.43, 1.79)	0.63 (0.32, 1.22)	0.88 (0.42, 1.86)	0.52 (0.12, 2.25)
aRR ^a^	0.92 (0.28, 3.02)	0.94 (0.29, 3.08)	0.93 (0.29, 3.05)	0.69 (0.32, 1.46)	0.63 (0.32, 1.23)	0.81 (0.38, 1.76)	0.59 (0.13, 2.63)
Early term birth							
cRR	0.99 (0.71, 1.38)	0.98 (0.70, 1.36)	0.99 (0.71, 1.38)	1.12 (0.91, 1.39)	1.06 (0.84, 1.34)	1.02 (0.62, 1.68)	1.02 (0.66, 1.56)
aRR ^a^	0.96 (0.69, 1.33)	0.95 (0.69, 1.32)	0.96 (0.69, 1.32)	1.09 (0.88, 1.34)	1.04 (0.83, 1.31)	1.05 (0.64, 1.71)	0.95 (0.63, 1.45)
Macrosomia							
cRR	2.24 (1.27, 3.97) **	2.25 (1.27, 4.01) **	2.24 (1.27, 3.97) **	0.96 (0.57, 1.60)	1.15 (0.66, 2.01)	2.32 (1.04, 5.19) *	2.58 (1.16, 5.72) *
aRR ^a^	2.11 (1.16, 3.83) *	2.22 (1.22, 4.03) *	2.19 (1.21, 3.98) *	0.90 (0.53, 1.53)	1.14 (0.64, 2.02)	2.45 (1.08, 5.57) *	2.50 (1.10, 5.68) *
LBW birth							
cRR	1.10 (0.34, 3.58)	1.20 (0.37, 3.93)	1.10 (0.34, 3.57)	0.50 (0.19, 1.32)	0.71 (0.32, 1.55)	0.63 (0.24, 1.67)	1.03 (0.29, 3.71)
aRR ^a^	1.13 (0.34, 3.74)	1.21 (0.36, 4.01)	1.14 (0.35, 3.74)	0.53 (0.20, 1.41)	0.70 (0.32, 1.57)	0.68 (0.25, 1.83)	1.03 (0.28, 3.71)
LGA birth							
cRR	1.25 (0.87, 1.80)	1.26 (0.87, 1.81)	1.25 (0.87, 1.80)	0.96 (0.73, 1.25)	0.85 (0.65, 1.11)	0.93 (0.49, 1.77)	1.03 (0.64, 1.67)
aRR ^b^	1.23 (0.86, 1.76)	1.24 (0.87, 1.78)	1.25 (0.87, 1.79)	0.96 (0.74, 1.26)	0.84 (0.65, 1.09)	0.93 (0.49, 1.75)	0.99 (0.62, 1.59)
SGA birth							
cRR	0.80 (0.29, 2.18)	0.90 (0.33, 2.45)	0.80 (0.29, 2.17)	0.39 (0.18, 0.85)	0.86 (0.47, 1.57)	0.45 (0.21, 1.00)	0.89 (0.30, 2.60)
aRR ^b^	0.72 (0.26, 1.96)	0.83 (0.30, 2.29)	0.72 (0.26, 1.97)	0.40 (0.18, 0.89) *	0.83 (0.45, 1.52)	0.48 (0.22, 1.07)	0.75 (0.26, 2.22)

Abbreviations: GDM, gestational diabetes mellitus; FA, folic acid; LBW, low birth weight; LGA, large-for-gestational-age; SGA, small-for-gestational-age. Data are presented as relative risk (95% confidence interval). Generalized linear models were used to assess the effects. Level of significance: * *p* < 0.05, ** *p* < 0.01. ^a^ Compared with cRR, aRRs were the effects adjusting for all potential confounders. ^b^ Here aRRs were the effects not adjusting for infant gender. ^c^ Model 1 assessed the effects of GDM on adverse birth outcomes. ^d^ Model 2 and Model 3 respectively adjusted for duration of FA supplementation before and during pregnancy based on Model 1. ^e^ Model 4 and Model 5 respectively assessed the effects of FA supplementation for ≥3 months before and during pregnancy on adverse birth outcomes adjusting for GDM. ^f^ Model 6 and Model 7 respectively assessed the interactions between GDM and the duration of FA supplementation before and during pregnancy on adverse birth outcomes.

**Table 5:** Associations between GDM and adverse birth outcomes stratified by duration of FA supplementation.

Outcomes	Total	FA Supplementation Before Pregnancy			FA Supplementation During Pregnancy		
		<3 Months	≥3 Months	MH Procedure	<3 Months	≥3 Months	MH Procedure
Cesarean delivery							
Model 1 ^a^	1.28 (1.02, 1.59) *	1.20 (0.88, 1.63)	1.37 (0.99, 1.90)	1.53 (1.00, 2.33)	1.25 (0.80, 1.93)	1.29 (0.99, 1.67)	1.53 (1.01, 2.34) *
Model 2 ^b^	1.30 (1.03, 1.63) *	1.20 (0.88, 1.63)	1.38 (0.95, 1.99)		1.19 (0.76, 1.86)	1.36 (1.06, 1.75) *	
Pre-term birth							
Model 1	0.80 (0.25, 2.56)	1.43 (0.44, 4.61)	−	0.81 (0.25, 2.67)	0.74 (0.10, 5.48)	0.83 (0.20, 3.48)	0.79 (0.24, 2.64)
Model 2	0.92 (0.28, 3.02)	1.89 (0.55, 6.48)	−		0.73 (0.07, 7.93)	0.97 (0.22, 4.20)	
Early term birth							
Model 1	0.99 (0.71, 1.38)	1.04 (0.68, 1.61)	0.90 (0.54, 1.50)	0.97 (0.61, 1.54)	1.17 (0.64, 2.15)	0.94 (0.63, 1.38)	0.99 (0.62, 1.57)
Model 2	0.96 (0.69, 1.33)	1.09 (0.71, 1.69)	0.96 (0.55, 1.68)		1.37 (0.65, 2.88)	0.89 (0.60, 1.31)	
Macrosomia							
Model 1	2.24 (1.27, 3.97) **	2.07 (0.97, 4.42)	2.56 (1.04, 6.29) *	2.46 (1.28, 4.73) **	2.23 (0.68, 7.37)	2.24 (1.17, 4.30) *	2.43 (1.27, 4.66) **
Model 2	2.11 (1.16, 3.83) *	1.99 (0.88, 4.50)	2.41 (0.90, 6.47)		1.92 (0.47, 7.80)	2.11 (1.06, 4.20) *	
Low birth weight							
Model 1	1.10 (0.34, 3.58)	1.73 (0.53, 5.66)	−	1.20 (0.36, 4.04)	−	1.75 (0.52, 5.90)	1.10 (0.33, 3.73)
Model 2	1.13 (0.34, 3.74)	1.76 (0.50, 6.15)	−		−	1.47 (0.41, 5.28)	
LGA birth							
Model 1	1.25 (0.87, 1.80)	1.51 (0.99, 2.32)	0.90 (0.46, 1.76)	1.34 (0.83, 2.18)	1.40 (0.75, 2.60)	1.19 (0.76, 1.86)	1.34 (0.82, 2.17)
Model 3 ^c^	1.23 (0.86, 1.76)	1.53 (0.99, 2.36)	1.01 (0.52, 2.00)		1.30 (0.66, 2.57)	1.15 (0.74, 1.78)	
SGA birth							
Model 1	0.80 (0.29, 2.18)	1.18 (0.44, 3.20)	−	0.89 (0.31, 2.53)	−	1.17 (0.42, 3.22)	0.79 (0.28, 2.25)
Model 3	0.72 (0.26, 1.96)	1.11 (0.40, 3.09)	−		−	0.96 (0.35, 2.65)	

Abbreviations: GDM, gestational diabetes mellitus; FA, folic acid; MH, Mantel-Haenszel; LGA, Large-for-gestational-age; SGA, Small-for-gestational-age. Data are presented as relative risk (95% confidence interval). Generalized linear models and Mantel-Haenszel chi-square tests were used to assess these effects. All relative risks in different groups were homogeneous by the Breslow-Day test. “-” means relative risk and P-value cannot be computed, due to the limited number of positive results of GDM and birth outcomes. Level of significance: * *p* < 0.05, ** *p* < 0.01. ^a^ Model 1 did not adjust for anything. ^b^ Model 2 adjusted for all potential confounders. ^c^ Model 3 included all confounders of Model 2 except for infant gender.

## Supplementary Materials

The following are available online at https://www.mdpi.com/1660-4601/16/22/4511/s1, Table S1: Maternal characteristics with missing values according to the duration of folic acid supplementation.
