# Supplementary material for: The Associations between the Duration of Folic Acid Supplementation, Gestational Diabetes Mellitus, and Adverse Birth Outcomes based on a Birth Cohort
Source: Int J Environ Res Public Health. 2019 Nov 15;16(22):4511. doi: 10.3390/ijerph16224511 (PMC6888242; doi:10.3390/ijerph16224511)
Supplement: Supplementary file 1 [file ijerph-16-04511-s001.pdf]

Table S1. Maternal characteristics with missing values according to the duration of folic acid supplementation.

Characteristics	Total	Before pregnancy		*p*-Value	During pregnancy		*p*-Value
		<3 months (n = 617) [a]	≥3 months (n = 280) [a]		<3 months (n = 229) [a]	≥3 months (n = 667) [a]	
Age at pregnancy (years)				0.059			0.462
< 25	45(5.0)	35(5.7)	10(3.6)		12(5.3)	33(5.0)	
25–29	421(47.1)	303(49.3)	118(42.3)		114(50.0)	306(46.0)	
30–34	317(35.5)	208(33.8)	109(39.1)		71(31.1)	246(37.0)	
≥ 35	111(12.4)	69(11.2)	42(15.1)		31(13.6)	80(12.0)	
Education				0.012 *			0.009*
≤Junior high	32(3.6)	28(4.6)	4(1.4)		15(6.6)	17(2.6)	
Senior high	111(12.6)	84(13.8)	27(9.8)		33(14.6)	78(11.9)	
≥College	740(83.8)	495(81.5)	245(88.8)		178(78.8)	561(85.5)	
Average monthly household income (CNY)				0.147			0.005 *
≤ 2000	29(3.3)	18(3.0)	11(4.1)		11(5.0)	18(2.8)	
2001–5000	465(53.5)	335(55.6)	130(48.7)		135(60.8)	330(51.1)	
> 5000	375(43.2)	249(41.4)	126(47.2)		76(34.2)	298(46.1)	
Postpartum BMI (kg/m^2)				0.638			0.775
< 18.5	15(1.8)	10(1.7)	5(1.9)		4(1.8)	11(1.7)	
18.5–23.9	490(57.2)	345(58.5)	145(54.5)		125(57.6)	365(57.1)	
24–27.9	277(32.4)	188(31.9)	89(33.5)		66(30.4)	211(33.0)	
≥ 28	74(8.6)	47(8.0)	27(10.2)		22(10.1)	52(8.1)	
Parity				0.317			0.366
Primiparous	622(69.7)	422(68.6)	200(71.9)		154(67.2)	467(70.4)	
Multiparous	271(30.3)	193(31.4)	78(28.1)		75(32.8)	196(29.6)	
Iron supplementation				0.003 *			0.005*
Yes	563(63.6)	369(60.4)	194(70.8)		127(55.9)	436(66.4)	
No	322(36.4)	242(39.6)	80(29.2)		100(44.1)	221(33.6)	
Multivitamin use				0.001*			0.107
Yes	523(59.1)	339(55.5)	184(67.2)		124(54.6)	399(60.7)	
No	362(40.9)	272(44.5)	90(32.8)		103(45.4)	258(39.3)	
Family history of diabetes				0.356			0.173
Yes	37(4.1)	28(4.5)	9(3.2)		13(5.7)	24(3.6)	
No	860(95.9)	589(95.5)	271(96.8)		216(94.3)	643(96.4)	
Gestational hypertension				0.351			0.077
Yes	44(5.1)	33(5.6)	11(4.1)		16(7.4)	28(4.3)	
No	817(94.9)	558(94.4)	259(95.9)		200(92.6)	616(95.7)	
Gestational anemia				0.089			0.081
Yes	115(12.8)	87(14.1)	28(10.0)		37(16.2)	78(11.7)	
No	782(87.2)	530(85.9)	252(90.0)		192(83.8)	589(88.3)	

Data are presented as frequency (%). The chi-square test was used to compare proportions. Level of significance: * $p < 0.05$. [a] Values for some characteristics may not be equal to the total numbers of 2 groups because of missing values.
